# Thymidine phosphorylase promotes malignant progression in hepatocellular carcinoma through pentose Warburg effect

**DOI:** 10.1038/s41419-018-1282-6

**Published:** 2019-01-17

**Authors:** Qiang Zhang, Yuan Qin, Jianmin Zhao, Yuanhao Tang, Xuejiao Hu, Weilong Zhong, Mimi Li, Shumin Zong, Meng Li, Honglian Tao, Zhen Zhang, Shuang Chen, Huijuan Liu, Lan Yang, Honggang Zhou, Yanrong Liu, Tao Sun, Cheng Yang

**Affiliations:** 1 State Key Laboratory of Medicine Chemical Biology and College of Pharmacy, Nankai University, Tianjin, China; 2 Tianjin Key Laboratory of Molecular Drug Research, Tianjin International Joint Academy of Biomedicine, Tianjin, China; 3 Department of Pathology, Beijing Friendship Hospital, Capital Medical University, Beijing, China; 4 College of Life Science, Nankai University, Tianjin, China

## Abstract

Tumor progression is dependent on metabolic reprogramming. Metastasis and vasculogenic mimicry (VM) are typical characteristics of tumor progression. The relationship among metastasis, VM, and metabolic reprogramming remains unclear. In this study, we identified the novel role of Twist1, a VM regulator, in the transcriptional regulation of thymidine phosphorylase (TP) expression. TP promoted the extracellular metabolism of thymidine into ATP and amino acids through the pentose Warburg effect by coupling the pentose phosphate pathway and glycolysis. Moreover, Twist1 relied on TP-induced metabolic reprogramming to promote hepatocellular carcinoma (HCC) metastasis and VM formation mediated by VE–Cad, VEGFR1, and VEGFR2 in vitro and in vivo. The TP inhibitor tipiracil reduced the effect of TP on promoting HCC VM formation and metastasis. Hence, TP, when transcriptionally activated by Twist1, promotes HCC VM formation and metastasis through the pentose Warburg effect and contributes to tumor progression.

## Introduction

Hepatocellular carcinoma (HCC) is a leading cause of cancer-related deaths worldwide; patients with HCC die mainly due to vasculogenic mimicry (VM) formation and metastasis^1–3^. The malignant progression of HCC is a response to the deterioration of the local tumor microenvironment. Blood supply is required to sustain tumor growth and metastasis. VM is a de novo microvascular channel formed by aggressive cancer cells and enables fluid transport from leaky vessels^4^. The pathways involved in VM formation share components with stemness and epithelial–mesenchymal transition (EMT), which are key attributes that promote tumor metastasis^5,6^. However, the mechanism by which tumor cells trigger VM formation remains unclear.

Under a deteriorated local tumor microenvironment, tumor cells are forced to reprogram cellular metabolism^7^. An obvious change in metabolism is the Warburg effect, where tumor cells mainly use glycolysis to generate energy even under aerobic conditions^8^. This metabolic reprogramming eliminates the threat of hypoxia to the survival of tumor cells. Under a nutrient-poor environment, tumor cells may preferentially utilize glutamine as a source of nutrients^9^. Moreover, tumor cells can use other carbon sources, such as lactate, serine, and glycine, as fuel^10–12^. By inducing cellular autophagy in paracancerous tissues, starved cancer cells can obtain fuel from extracellular sources^13^. Distant metastases depend on the pentose phosphate pathway for reprogramming malignant gene expression and phenotype^14^. These metabolic reprogramming processes could prevent tumor cells from surviving stress before VM formation. However, whether other metabolic reprogramming is involved in tumor malignant progression before VM formation and whether this metabolic reprogramming is related to VM formation and metastasis remain unclear. Thus further explorations are required.

Twist1 is a key transcription factor that induces EMT and VM by upregulating VE–cadherin expression^15^. Twist1 transcriptionally promotes the expression of thymidine phosphorylase (TP), also known as platelet-derived endothelial cell growth factor^16^. When tumor vascular supply is occluded, TP exhibits high expression under a hypoxic and low-pH environment^17^. As a phosphorylase, TP catalyzes the conversion of thymidine into deoxyribose-1-phosphate (dR-1-P), which is then converted into dR-5-P, glyceraldehyde-3-phosphate (G-3-P), or deoxyribose^18^. TP promotes endothelium-dependent angiogenesis in endothelial cells^19^. A previous study demonstrated that TP promotes metastasis and is a poor prognostic marker in HCC^20^. In the present study, we explored whether TP upregulation affects the metabolic reprogramming of HCC and whether the transcriptional pattern of Twist1–TP could contribute to VM formation in HCC.

## Materials and methods

### Case selection

HCC tissue microassays of 306 cases were purchased from US Biomax for immunohistochemical (IHC) or PAS&CD31 double staining and for analysis of correlation among metastasis, clinical stage, pathology grade, carcinoembryonic antigen (CEA) content, alpha-fetoprotein (AFP) content, gender, survival time, VM formation, and expression of VE–cadherin, vascular endothelial growth factor receptor 1 (VEGFR1), VEGFR2, Twist1, and TP. HCC characteristics were categorized based on the best cut-off values or staining index.

### The Cancer Genome Atlas (TCGA) data analysis

The genomic data of cancer cases were downloaded from TCGA. Differentially expressed genes were screened based on a |log2fold change| ≥ 0.7. The co-expressed genes of Twist1 were analyzed, and genes with co-expression Pearson coefficient >0.3 were considered co-expressed with Twist1. The top 10% of the co-expressed genes of Twist1 were screened. The co-expressed genes of Twist1 were Venn analyzed with the chromatin immunoprecipitation–sequencing (ChIP-seq) results.

### Chromatin immunoprecipitation–sequencing

In brief, 1–1.5 × 10^7^ cells were cross-linked with 1% formaldehyde (Sigma, USA) for 10 min, quenched with 0.25 M glycine, and washed in cold phosphate-buffered saline. The cells were incubated with the ChIP lysis buffer containing the protease inhibitor of cocktail (Roche, Switzerland). The extracted chromatin was sheared to an average length of 200–400 bp with micrococcal nuclease. The chromatin fraction was incubated with Twist1 antibody (1:100, Abcam) overnight at 4 °C. The protein/DNA complexes were reversed cross-linked to obtain free DNA. DNA fragments were isolated by agarose gel purification, ligated to primers, and subjected to Solexa sequencing according to the manufacturer’s recommendations (Illumina Inc., USA). Sequence information was analyzed using the HG18 annotation database.

### IHC analysis

IHC was performed to detect the expression levels of different proteins. Tissue sections were deparaffinized in xylene and rehydrated by gradient alcohol prior to IHC. Endogenous peroxidase activity was blocked by incubation with 3% hydrogen peroxide in methanol for 30 min. The tissue sections were heated using 0.01 M citric acid buffer for 10 min in a microwave oven. The slides were stained with antibodies against Twist1 (1:100, Santa Cruz Biotechnology), TP (1:100, Abcam), VE–cad (1:50, Abcam), VEGFR1 (1:250, Abcam), VEGFR2 (1:50, Abcam), or CD31 (1:50, Abcam). After incubation with horseradish peroxidase-conjugated goat anti-rabbit/mouse immunoglobulin G (IgG), the sections were stained with 3,3′-diaminobenzidine and counterstained with hematoxylin or periodic acid–Schiff (PAS). Finally, the sections were dehydrated and mounted. IHC staining was scored by multiplying the positive degree (0 for none, 1 for weak brown, 2 for moderate brown, and 3 for strong brown) and positive rate (1 for 0–25%, 2 for 25%–50%, 3 for 50%–75%, and 4 for 75%–100%). Sections with staining indices >6 were classified to the high-expression group. PAS-positive channels without CD31 staining were classified to the VM group. Five random ×40 fields per HCC tissue were scored with morphology consistent with mimicry. All quantification experiments were performed in a blinded setting.

### Plasmids

Plasmids of pCDNA3.1-Twist1, pRNAT-shTwist1, M02, M02-TP, psiU6, and psiU6-TP were purchased from GeneCopoeia (Guangzhou, China). The reporter gene plasmid PGL4.3-TP and its truncations and mutations were constructed by inserting the TP promoter regions that were PCR-amplified from human genomic DNA into the PGL4.3 vector (Table S1).

### Cell culture and transfection

Human HCC cell lines, namely, SNU387, PLC-PRF-5, Hep3B, HepG2, Huh-7, MHCC-9L, and MHCC-97H, were obtained from the American Type Culture Collection and KeyGen Biotech (Nanjing, China). The HCC cells were cultured in RPMI-1640 medium or Dulbecco’s modified Eagle’s medium (DMEM; Hyclone) with 10% fetal bovine serum (FBS; Hyclone). Glucose-free medium RPMI-1640 (Life Technologies, China) and DMEM (Neuronbc, China) were used for starvation experiments, added with 100 nM thymidine (Amresco, USA), and further incubated. TP enzyme inhibitor tipiracil (TPI; 10 μM, Meilunbio, China) was added to the medium for enzyme inhibition experiments. The vectors were transfected into cells by using transfection reagents (Roche, Switzerland).

### Western blot analysis

Immunoblots were obtained from samples containing 30 μg of the total protein through sodium dodecyl sulfate-polyacrylamide gel electrophoresis and transferred onto polyvinylidene difluoride membranes (Millipore, USA). The membrane was incubated with primary antibodies against Twist1 (Santa Cruz Biotechnology, USA), TP (Abcam, UN), VE–cadherin (Affinity Bioreagents, USA), VEGFR1 (Affinity Bioreagents, USA), VEGFR2 (Affinity Bioreagents, USA), or glyceraldehyde 3-phosphate dehydrogenase (GAPDH; Affinity Bioreagents, USA), followed by secondary antibody (Santa Cruz Biotechnology, USA). Blots were detected using an enhanced chemiluminescence detection kit (Millipore, USA). Densitometric analysis was performed using the ImageJ software. The ratio of densitometry value to the corresponding GAPDH value was used to indicate the relative protein expression.

### Luciferase assays

After transfection of the reporter gene plasmids, the cells were cultured for another 48 h. Transactivation assays were performed with Dual-Luciferase Assay System (Promega), and luciferase activities were measured using the Luminoskan Ascent reader system (Thermo Scientific, USA).

### RNA-seq and omics analysis

PLC-PRF-5 cells transfected with control plasmids, pCDNA3.1–Twist1, or M02–TP; added with thymidine (dT); or added with dT after transfection of M02-TP were collected with TRIzol (Sigma, USA) for RNA isolation. After preparation of biotinylated complementary RNA (cRNA) by using the Illumina TotalPrep RNA Amplification Kit (Ambion), cRNA was hybridized with BeadChip. The samples were prepared as technical duplicates. Data were acquired with Illumina BeadChip Reader and evaluated using Illumina BeadStudio Application by Genergy Bio (China). Gene set enrichment analysis (GSEA)^21^ and gene ontology analysis were used to detect changes in biological processes, cellular components, and molecular functions. Pathway analysis was conducted based on the KEGG database. Protein–protein interaction (PPI) was analyzed using the STRING database^22^ and Cytoscape software (NRNB)^23^.

### Enzyme-linked immunosorbent analysis (ELISA)

Commercial TP ELISA kit (R&D, USA) was used to measure TP concentration in the culture medium in accordance with the manufacturer’s instructions. Medium samples from cultures in exponential growth were collected 5, 24, and 48 h after change of medium. Concentrated medium was examined diluted 200×. Each experiment was repeated in triplicate, and mean values (mean ± SD) are presented.

### Metabolite detection

The metabolite of ATP was detected using the ATP Assay Kit (Beyotime, China). The metabolites of NADH and NADPH were detected using the NAD^+^/NADH and NADP^+^/NADPH assay kits (BioAssay Systems, USA). Other metabolites in the medium or PLC-PRF-5 cells treated with dT and overexpressed with or without TP were detected by gas chromatography (GC) time of flight mass spectrometry (MS).

### Wound healing assay

The transfected cells were seeded into 24-well culture plates at a density of 5 × 10^5^ cells/well. Tumor cells adhering to the plate were removed by scratching the wound with a 200 µL pipette tip and incubated in serum-free medium for 24 h. Images of the wounds were taken under a light microscope (Nikon, Japan). Each experiment was repeated in triplicate, and mean values ± SD are presented.

### Matrigel invasion assay

Cell invasion assay was performed using Transwell cell culture inserts (Corning, USA) and Matrigel (BD, USA). The transfected cells were allowed to invade for 24 h. The passed cells were fixed in 4% paraformaldehyde, stained with crystal violet solution, and then counted under a light microscope (Nikon, Japan).

### Three-dimensional culture assay

PLC-PRF-5 and Hep3B cells transfected with different plasmids were seeded into a 48-well plate precoated with Matrigel and cultured in 250 µL of RPMI-1640 medium or DMEM containing 10% FBS for 24 h. Images of the tube were taken under a light microscope (Nikon, Japan). The tube structures were assessed to evaluate the ability of tube formation. Other samples were seeded on climbing slides; fixed with glutaraldehyde, osmic acid, and tert-butyl alcohol; coated with a thin layer of gold; and then imaged under a scanning electron microscope (SEM; LEO 1503 VP, Germany).

### Immunofluorescence staining

The transfected cells were plated into 96-well plates at a density of 4000 cells/well. The cells were fixed with ice-cold methanol and blocked with serum. The primary antibodies of VEGFR1, VEGFR2, and VE–Cad (Abcam, USA) were used at a 1:100 work solution. Fluorescein isothiocyanate- and tetramethyl rhodamine isothiocyanate-conjugated mouse and rabbit IgG antibodies (Santa Cruz Biotechnology, USA) were used to label immunofluorescence. After immunolabeling, the cells were stained with Hoechst (Sigma, Germany) and measured with high-content screening (HCS) systems to evaluate the protein expression (Thermo Scientific, USA).

### Molecular docking

Molecular docking was performed using the Schrodinger software. The crystal structure of TP was downloaded from the PDB database (https://www.rcsb.org, PDB code: 2WK6). The ligand in TP was extracted from the crystal structure as the location center of docking. The structure of TPI was generated by Chemdraw and optimized by the LigPrep module of Schrodinger. The Glide XP (extra precision) mode was used for docking score calculation.

### Animal studies

Twenty BALB/c nu/nu mice aged 5–6 weeks (equal amount of males and females) were divided randomly into five groups. PLC-PRF-5 cells transfected with control plasmids, pCDNA3.1-Twist1, M02-TP, pCDNA3.1-Twist1, and M02-TP or pCDNA3.1-Twist1 and psiU6-TP were subcutaneously injected into the flank in separate groups at a density of 5 × 10^6^ cells/mouse. After tumor inoculation, tumor size was measured every 2 days for another 18 days. In the enzyme inhibition experiments, the tumor-bearing mice were orally treated with TPI at a dose of 45 mg/kg/day every 2 days for 26 days. All mice were sacrificed, and xenografts and lungs were collected. Lung tissues were harvested for histologic examination to measure the extent of metastasis, and the xenografts were further analyzed by IHC and PAS&CD31 double staining.

### Statistical analysis

Differences among the groups were analyzed by one-way analysis of variance with Bonferroni post hoc test. Qualitative variables were analyzed by Fisher’s exact two-tailed test or chi-squared test. Overall survival analysis was performed using Kaplan–Meier method with the log-rank test. All data in the study were evaluated with the IBM SPSS Statistics 22.0 software (Chicago, IL, USA). *P* values <0.05 were deemed statistically significant.

## Results

### Twist1 promotes TP transcription by binding to the conserved motifs in the promoter region

**Fig. 1:**
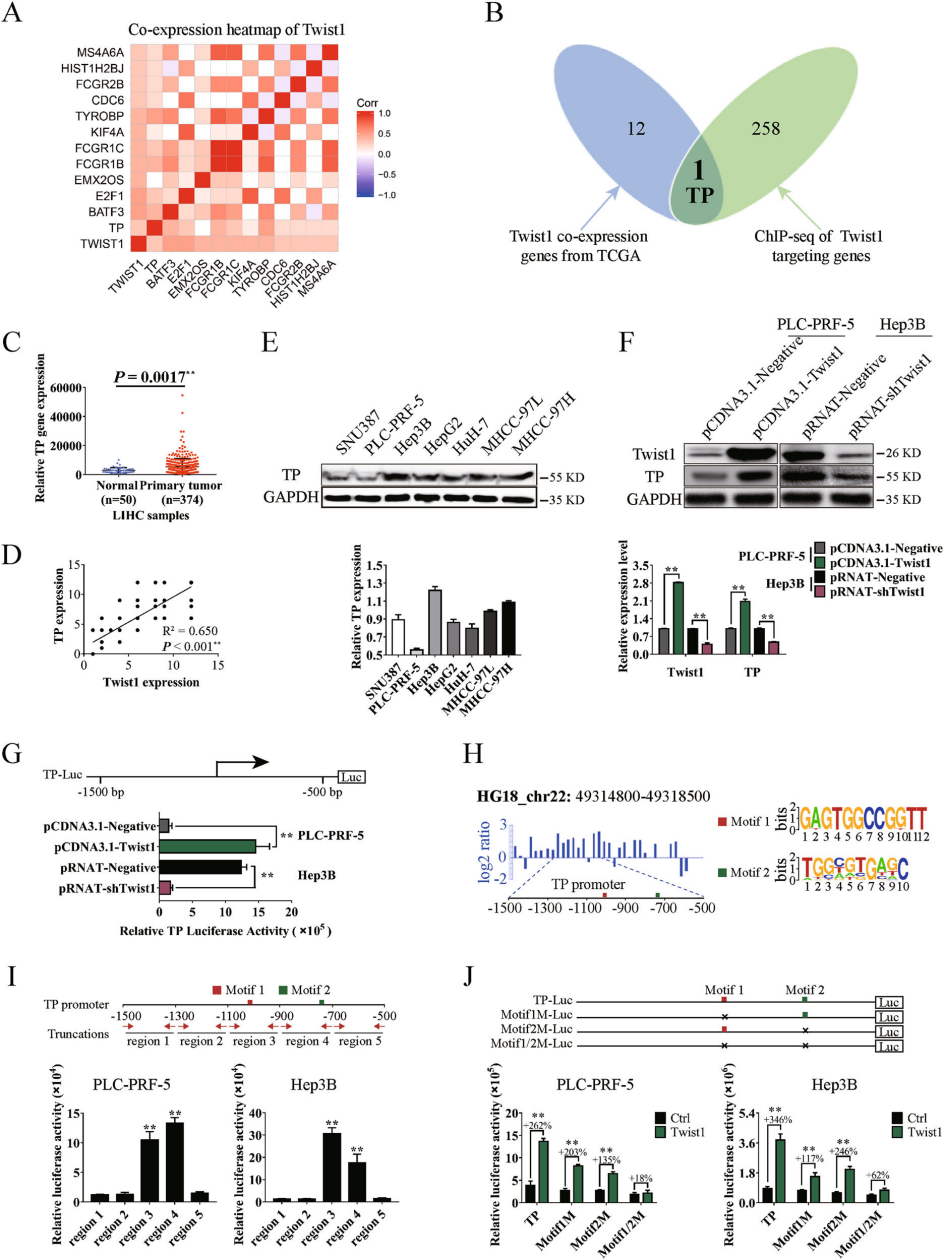
Twist1 promotes the transcription of the thymidine phosphorylase (TP) gene by binding to the conserved motifs in the promoter region. **a** Heat map of Twist1 co-expression genes downloaded from The Cancer Genome Atlas (TCGA) database. **b** Venn analysis for identifying Twist1 target genes in human hepatocellular carcinoma (HCC). The blue diagram represents Twist1 co-expression genes from TCGA, and the green diagram represents Twist1 targeting genes by chromatin immunoprecipitation–sequencing (ChIP-seq). **c** TP expression was significantly upregulated in LIHC samples compared with the normal liver tissues. **d** Correlation analysis of Twist1 and TP expression in clinical HCC specimens. **e** Relative TP expression analysis in seven HCC cell lines. The ratio of densitometry value to the corresponding glyceraldehyde 3-phosphate dehydrogenase (GAPDH) value was used to indicate relative protein expression. **f** Western blot used to analyze TP protein levels influenced by Twist1 in PLC-PRF-5 and Hep3B cells. **g** Twist1 promoted TP gene transcription as detected by luciferase assay. **h** Diagram of the ChIP-seq binding peaks related to the TP promoter. Binding site analysis showed two individual conserved motifs within the promoter of TP. **i** Luciferase assay results of Twist1 to the truncated TP promoter regions demonstrated that Twist1 bound to the TP promoters of region 3/4. **j** Luciferase assay results of Twist1 to the mutated TP promoter regions demonstrated that Twist1 bound to motif 1/2 in the TP promoter. (mean ± SD; *n* = 3 in triplicate; **P < 0.01)

The candidate co-expression genes with Twist1 from the TCGA database were screened. Thirteen genes were found to be significantly correlated with Twist1 (Fig. 1a). ChIP results were used to screen the transcriptional targets of Twist1. We defined genes by binding peaks in the promoter region. A total of 259 genes were targeted by Twist1 (Fig. 1b, green circle). By Venn analysis, we identified TP in both the predicted gene sets (Fig. 1b). Further analysis of the LIHC data downloaded from the TCGA database showed that the TP gene exhibited higher expression level in the primary HCC samples than in the normal liver samples (Fig. 1c). To illustrate the malignant progression effects of TP in HCC, we grouped the clinical tissue samples based on clinical parameters. Kaplan–Meier survival analysis results showed that the high TP expression significantly affected the survival prognosis of patients with HCC with clinical parameters of stage ≥II, metastasis or CEA content >3.4 ng/mL, AFP content >400 ng/mL, or alanine aminotransferase content >38 U/L (Figure S1).

To further prove the transcriptional activation of TP by Twist1, we analyzed the correlation between Twist1 expression and TP expression in the HCC samples. Results showed a significant correlation between these two proteins (Fig. 1d). Seven HCC cells were chosen to detect the background expression levels of TP. PLC-PRF-5 and Hep3B were separately used as TP low-expression and high-expression cell line models for subsequent experiments (Fig. 1e). Western blot results showed that TP expression was significantly promoted when the Twist1 expression was upregulated in PLC-PRF-5 cells and significantly reduced when Twist1 was knocked down in Hep3B cells (Fig. 1f). Based on the luciferase assay, the ectopic overexpression of Twist1 enhanced the transcriptional activation of TP in PLC-PRF-5 cells, and Twist1 downregulation reduced the activity in Hep3B cells (Fig. 1g). According to the peak regions from the ChIP-seq assay, Twist1 primarily recognized the 1500 bp upstream of the TP promoter transcription start sites (Fig. 1h, left panel). To further identify Twist1-binding sites, we reanalyzed the generic feature format data from the ChIP-seq assay by using the ChIPseeker online software^24^. Two individual motifs and their locations within the promoter of TP were found. Motif1 and Motif2 possessed the relative conserved sequences of GAGTGGCCGGTT and TGGCGTGAGC, respectively (Fig. 1h, right panel). The truncated and mutated luciferase report plasmids were used for motif identification. The TP promoter of the −1500 bp to −500 bp region was truncated to five shorter regions, in which region3 of −1100 bp to −900 bp and region4 of −900 bp to −700 bp contained the conserved motif (Fig. 1i, upper panel). The luciferase assay results showed that all Twist1 transcriptionally activating regions contained the conserved Motif1 or Motif2 (Fig. 1i, lower panel). Meanwhile, we constructed mutation plasmids with deletion mutations of Motif1 or/and Motif2. The Twist1 transcriptional activations were weakened when Motif1 or Motif2 was deleted. When both motifs were deleted, Twist1 transcriptional activation disappeared (Fig. 1j). These results indicate that both motifs are related to the transcription of TP.

### TP stimulates the pentose Warburg effect on HCC cells

**Fig. 2:**
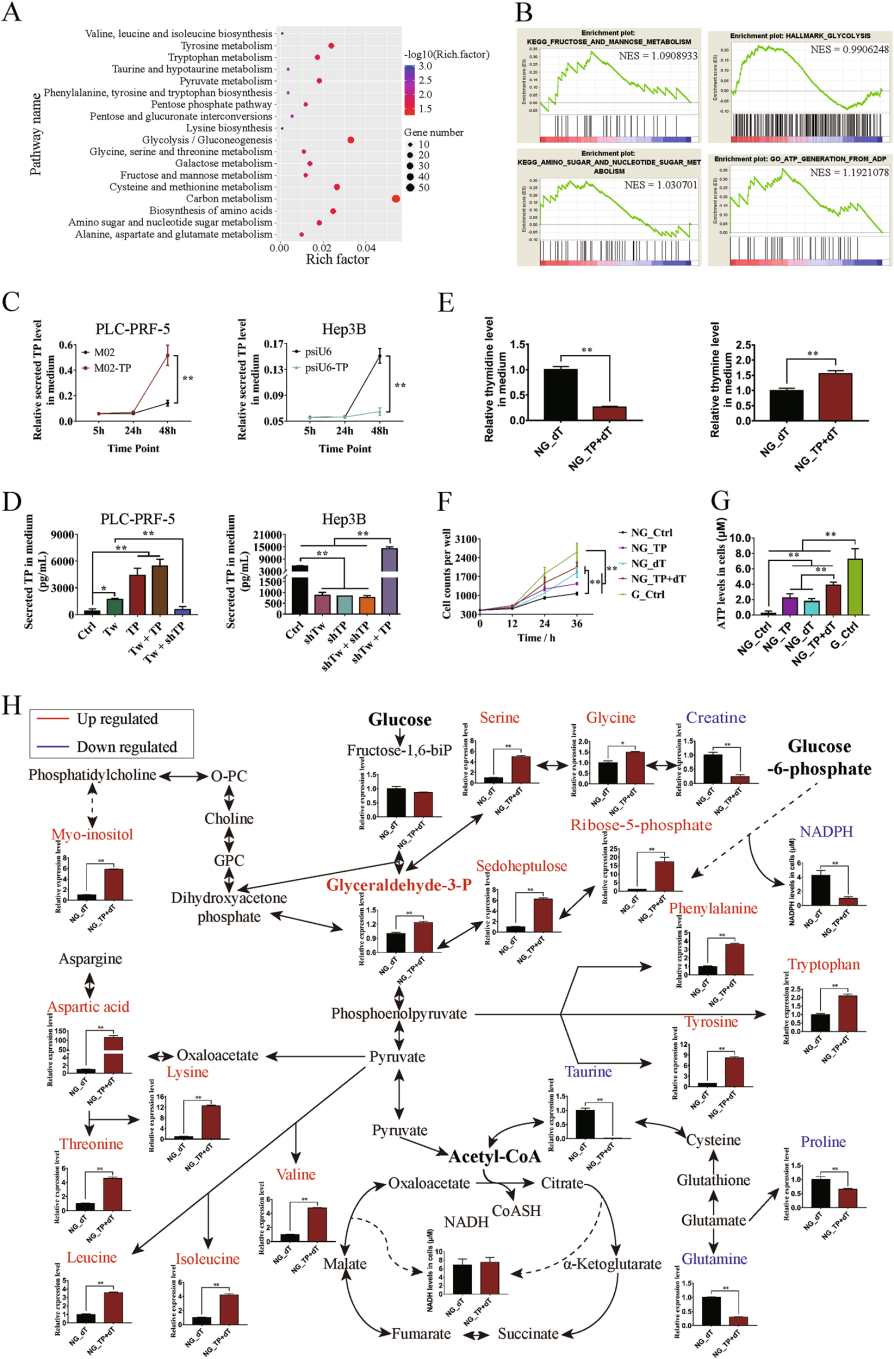
Thymidine phosphorylase (TP) stimulates the pentose Warburg effect on hepatocellular carcinoma (HCC) cells. **a** Overexpression of Twist1 activated a series of pathways related to glycometabolism and amino acid metabolism. **b** Enrichment analysis of the gene expression profiles in PLC-PRF-5 cells overexpressing Twist1 detected by gene set enrichment analysis. **c** Enzyme-linked immunosorbent assay was used to analyze the TP factor levels in the medium. Overexpression of TP promoted the amount of TP secreted in PLC-PRF-5 cells; knocking down of TP reduced the amount of TP secreted in Hep3B cells. **d** Correlation between Twist1/TP expression levels and secreted TP factor levels in PLC-PRF-5 and Hep3B cells. **e** Overexpression of TP promoted the enzymatic metabolism of extracellular dT in the medium without glucose. **f** Enzymatic metabolism of extracellular dT regulated by TP promoted HCC cell proliferation. **g** Enzymatic metabolism of extracellular dT regulated by TP promoted ATP generation in PLC-PRF-5 cells. **h** Enzymatic metabolism of extracellular dT regulated by TP affected glycometabolism and amino acid metabolism in glucose-free cultured PLC-PRF-5 cells. NG means “No Glucose.” (mean ± SD; *n* = 3 in triplicate; *P < 0.05; **P < 0.01)

RNA-seq was performed to study the potential functions of TP transcriptionally regulated by Twist1 in HCC cells. The pathway analysis results showed that Twist1 overexpression activated the pentose phosphate pathway, glycolysis, and the metabolism of a series of carbon compounds (Fig. 2a). The GSEA results further proved that Twist1 promoted fructose and mannose metabolic pathways, glycolysis, amino sugar nucleotide sugar metabolic pathway, and ATP generation from the ADP process (Fig. 2b). Although the role of secretion of TP was not explained, we suspected that the secreted TP might play a role in HCC metabolism reprogramming. The ELISA results showed that the secretion of TP factors in PLC-PRF-5 cells was significantly promoted by TP overexpression and inhibited by TP knockdown (Fig. 2c). When Twist1 or TP was upregulated in PLC-PRF-5 cells, the amount of secreted TP in the medium significantly increased. TP secretion was promoted when Twist1 and TP were upregulated. Knockdown of TP decreased the promotion of TP secretion by Twist1. Similarly, knockdown of Twist1 or/and TP significantly inhibited the secretion of TP in Hep3B cells (Fig. 2d).

When the microenvironment becomes deteriorated, some tumor cells die due to carbon source pressure and the dead tumor cells will release the remaining carbon sources, including thymidine. To study whether the secreted TP is an important enzyme for using extracellular thymidine as the sole carbon source of near-death tumor cells under the deteriorated microenvironment, we added thymidine to the medium without glucose. To confirm the enzyme activity of the secreted TP on extracellular thymidine, we analyzed the medium by GC–MS analysis. The amount of thymidine significantly decreased and that of thymine significantly increased in the medium (Fig. 2e). Proliferation activities were detected every 12 h for a total of 36 h to study the effects of TP on HCC cell proliferation. In the absence of glucose, the overexpression of TP or the addition of dT promoted the proliferation of PLC-PRF-5 cells, and the cell count significantly increased when dT was added after TP overexpression (Fig. 2f). The generation of ATP was then analyzed. In the absence of glucose, the overexpression of TP or the addition of dT significantly promoted ATP synthesis. The ATP levels were further increased when TP was upregulated with the addition of dT. When cultured with glucose-containing medium, PLC-PRF-5 cells generated the highest amount of ATP (Fig. 2g). Further analysis results showed that, in the absence of glucose, the overexpression of TP with the addition of dT did not promote the generation of NADH and NADPH. The generation of metabolic intermediates, including ribose-5-phosphate, sedoheptulose, and glyceraldehyde-3-phosphate, in the pentose phosphate pathway significantly increased. The contents of myo-inositol and amino acids including serine, glycine, phenylalanine, tryptophan, tyrosine, aspartic acid, lysine, threonine, valine, leucine, and isoleucine that were derived from the glycolysis pathway significantly increased. Meanwhile, the contents of taurine and amino acids, namely, proline and glutamine, derived from TCA significantly decreased (Fig. 2h). These results indicate that the main metabolic pathways affected by TP are the pentose phosphate pathway and glycolysis.

### Twist1 relies on TP to promote HCC cell migration, invasion, and VM formation through TP enzyme activities

**Fig. 3:**
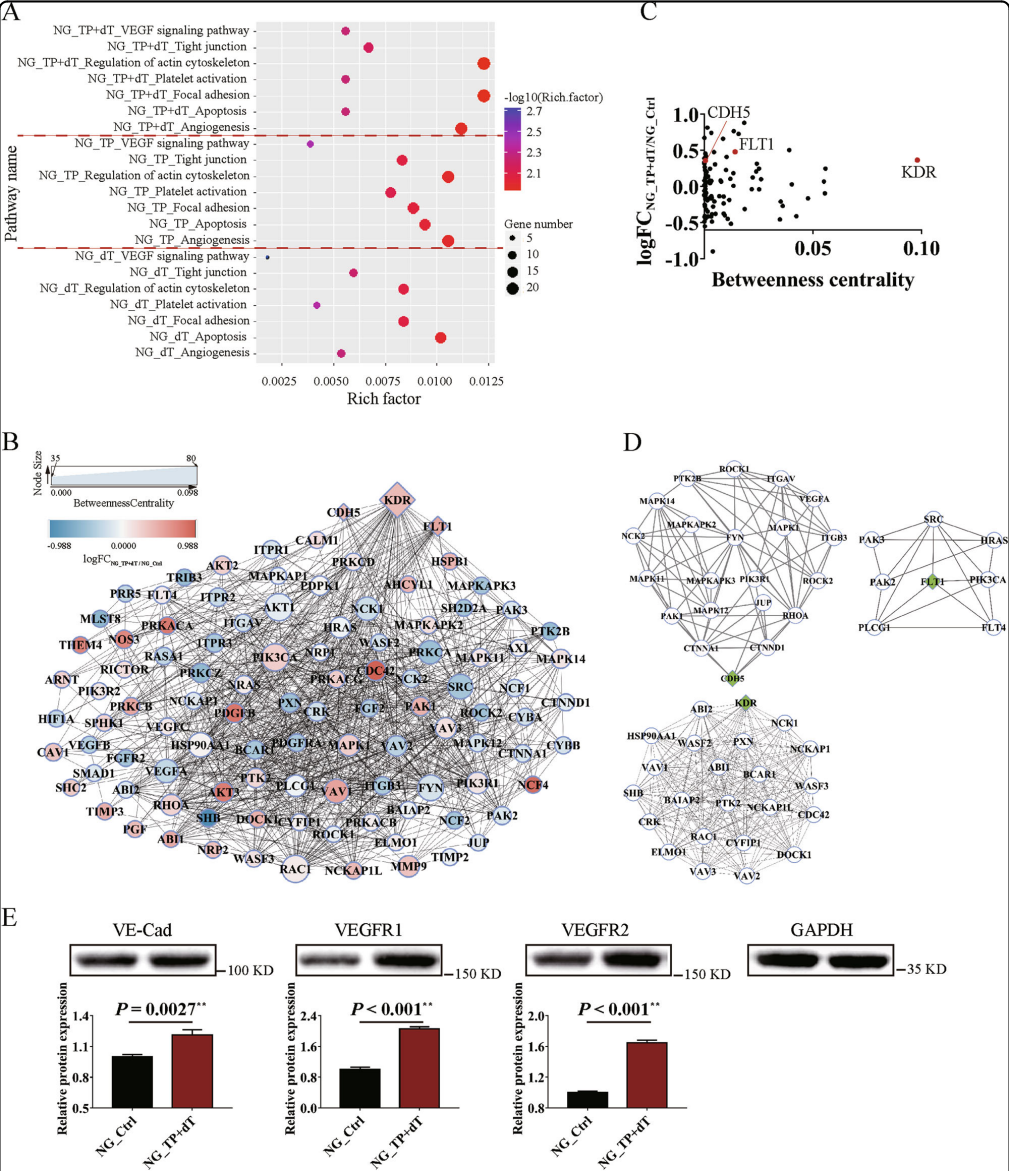
Enzymatic metabolism of extracellular dT regulated by thymidine phosphorylase (TP) affects tumor functions related to vasculogenic mimicry (VM) formation in hepatocellular carcinoma cells. **a** Pathway analysis of glucose-free cultured PLC-PRF-5 cells affected by overexpressing TP (NG_TP), adding dT (NG_dT), or overexpressing TP and adding dT (NG_TP + dT). **b** Protein–protein interaction (PPI) analysis of genes involved in the pathways affected by overexpressing TP and adding dT. Major hubs related to VM formation are highlighted with diamond boxes. **c** Analysis of network topology, including logFC and Betweenness Centrality, reveals VM formation-related PPI hubs. **d** PPI hub diagrams for VM formation-related genes, namely, CDH5, FLT1, and KDR. **e** Western blot analysis of the expression levels of VE–Cad, vascular endothelial growth factor receptor 1 (VEGFR1), and VEGFR2 influenced by overexpressing TP and adding dT in glucose-free cultured PLC-PRF-5 cells. The ratio of densitometry value to the corresponding glyceraldehyde 3-phosphate dehydrogenase (GAPDH) value was used to indicate the relative protein expression. NG means “No Glucose” (mean ± SD; *n* = 3 in triplicate; **P < 0.01)

The RNA-seq results of the different treated PLC-PRF-5 cells were analyzed to clarify the influence of the pentose Warburg effect regulated by TP enzyme activities on HCC malignant progression. In the absence of glucose, the overexpression of TP or the addition of dT affected a series of pathways related to angiogenesis and metastasis; such pathways included the VEGF signaling pathway, the tight junction pathway, and the actin cytoskeleton pathway. The influences on these pathways were more obvious when TP was upregulated with the addition of dT (Fig. 3a). The genes involved in these pathways were analyzed with PPI. Based on logFC and Betweenness Centrality index values for network nodes, tumor VM formation-related genes, namely, KDR, FLT1, and CDH5, were revealed to be important upregulated hub genes (Fig. 3b, c). The selected PPI hubs, namely, KDR, FLT1, and CDH5, with novel connectivity exhibited potentially critical biological roles in HCC VM formation (Fig. 3d). The western blot results showed that the corresponding proteins of VE–Cad, VEGFR1, and VEGFR2 were significantly upregulated when dT was added after TP overexpression compared with the control group (Fig. 3e).

**Fig. 4:**
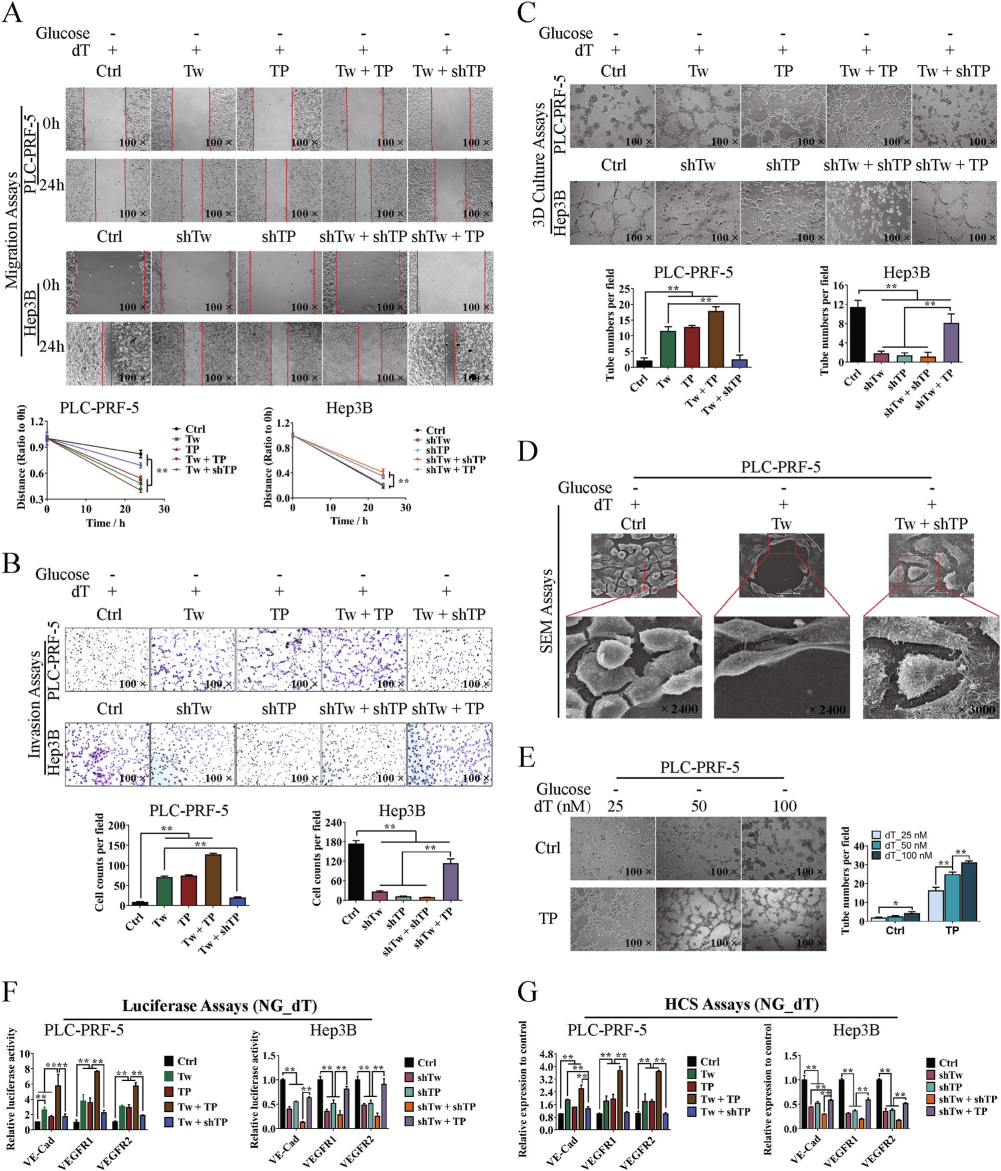
Twist1–thymidine phosphorylase (TP) transcriptional pattern promotes hepatocellular carcinoma cell migration, invasion, and vasculogenic mimicry (VM) formation through enzymatic metabolism of extracellular dT. PLC-PRF-5 cells were upregulated with Twist1 and/or TP or upregulated with Twist1 and knocked down of TP. Hep3B cells were knocked down of Twist1 and/or TP or knocked down of Twist1 and upregulated with TP. Cells were cultured in glucose-free medium added with the TP substrate of dT. **a** Wound healing assay showed a significant difference in the speed of cell migration among different treated groups. **b** Invasion assay showed a significant difference in the speed of cell invasion among different treated groups. **c** In vitro assay for VM in three-dimensional culture at 24 h. Upregulation of Twist1 and/or TP promoted the tube formation in PLC-PRF-5 cells, and knocking down of TP attenuated the promotion effect of Twist1. Knocking down of Twist1 and/or TP inhibited the tube formation in Hep3B cells, and upregulating TP significantly reduced the inhibitory effect of knocking down of Twist1. **d** Morphological observation of different treated PLC-PRF-5 cells in pseudopod and cell rounding. **e** TP relied on the extracellular dT to promote the tube formation of glucose-free cultured PLC-PRF-5 cells. **f** Transcriptional regulatory activities of Twist1 and TP on VE–Cad, vascular endothelial growth factor receptor 1 (VEGFR1), and VEGFR2. **g** Protein expression analysis of VE–Cad, VEGFR1, and VEGFR2 with the HCS systems. NG means “No Glucose” (mean ± SD; *n* = 3 in triplicate; *P < 0.05; **P < 0.01)

Considering that VM formation was associated with cell migration and invasion, we investigated the effect of Twist1/TP on cell migration, invasion, and VM formation in a three-dimensional (3D) culture system in vitro by using the upregulated cell model in PLC-PRF-5 and the knockdown cell model in Hep3B. Wound healing assay showed significant differences in wound healing speed in the transfected cells between the two cell models (Fig. 4a). The invasion ability increased about four times compared with the control group when Twist1 or TP was upregulated and about six times when Twist1 and TP were both upregulated. However, the invasion ability of PLC-PRF-5 cells significantly decreased when TP was knocked down even though Twist1 was upregulated. The invasion ability was lower than that in the control group when Twist1 was knocked down and TP was overexpressed in Hep3B cells but significantly increased when Twist1, TP, or both were downregulated (Fig. 4b). The 3D culture assay showed that the number of typical pipetube-like structures within the 3D Matrigel medium increased when Twist1 and/or TP were/was upregulated, while knocking down of Twist1 and/or TP decreased the tube formation. The network formation decreased to the control level when Twist1 was upregulated and TP was knocked down in PLC-PRF-5 cells. The tube formation was significantly enhanced when Hep3B cells were transfected with pRNAT-shTwist1 and M02-TP than when Twist1, TP, or both were knocked down in the cells (Fig. 4c). Based on the SEM results, the PLC-PRF-5 cells subjected to different treatments showed significant changes in morphology, including pseudopod disappearance and cell rounding (Fig. 4d). Gradient concentrations of dT were added to the glucose-free medium of control or TP-upregulated PLC-PRF-5 cells, and the tube formation was assessed. TP gradually promoted the tube formation as the dT concentration was increased from 25 to 100 nM (Fig. 4e). To further demonstrate the relation between Twist1–TP transcriptional pattern and VM formation, we determined the expression levels of VE–Cad, VEGFR1, and VEGFR2. After transfecting the luciferase reporter plasmids of VE–Cad, VEGFR1, and VEGFR2, the transcription of these marker genes was promoted when either Twist1 or TP was upregulated in PLC-PRF-5 cells. Meanwhile, the knockdown of TP impaired the transcriptional activity even though Twist1 was upregulated. The transcriptional activities of the marker genes were significantly decreased when Twist1 and/or TP were knocked down but were enhanced by M02-TP transfection (Fig. 4f). Through the immunofluorescence assay, we labeled the three marker proteins with fluorescence and analyzed them by using the HCS system. The protein expression levels of VE–Cad, VEGFR1, and VEGFR2 were consistent with their transcriptional levels (Fig. 4g).

### Twist1 relies on TP to promote VM formation and metastasis in vivo

**Fig. 5:**
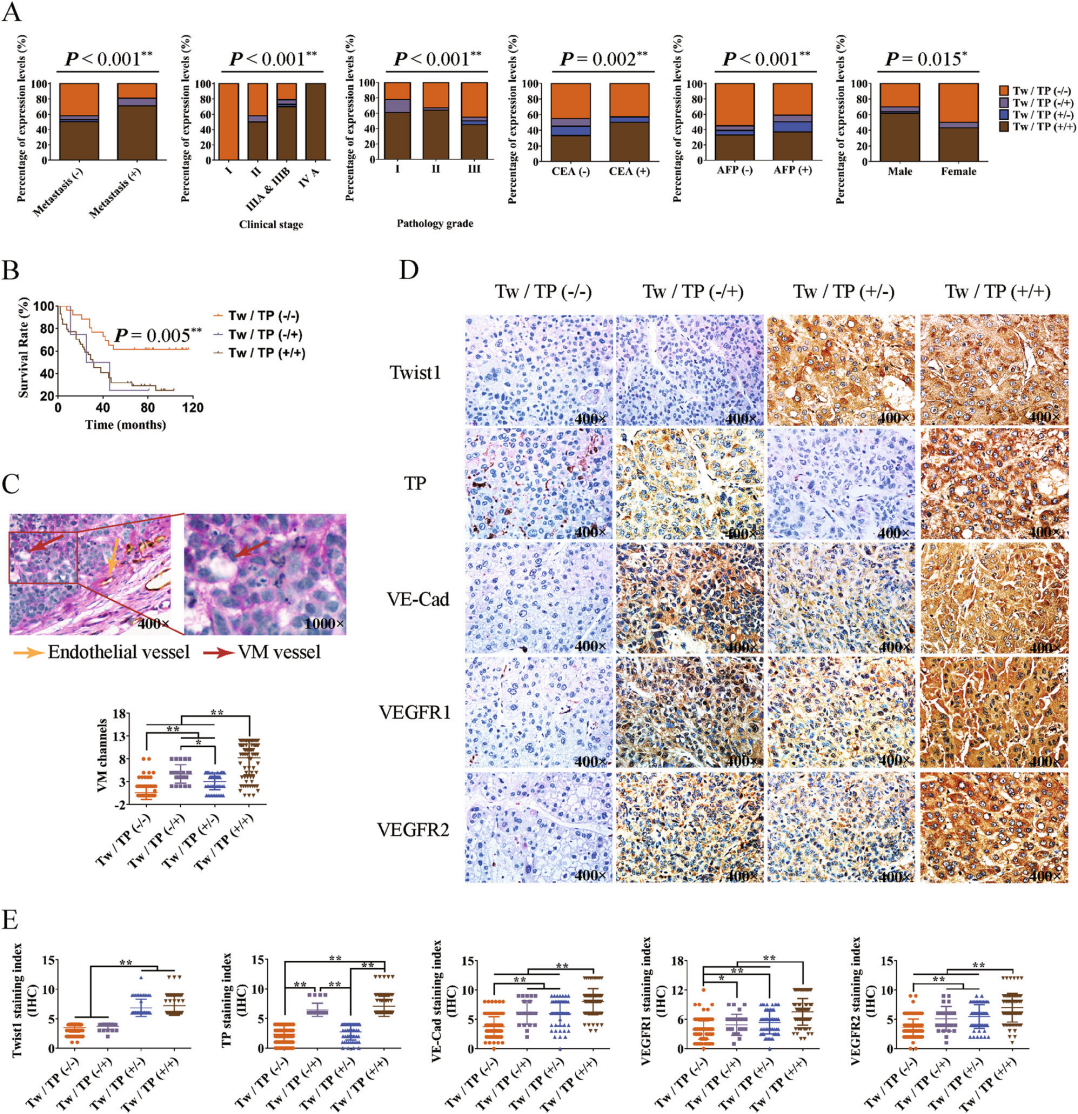
Twist1 relies on thymidine phosphorylase (TP) to promote hepatocellular carcinoma (HCC) malignant progression. HCC samples were divided into four groups of Twist1/TP (−/−), Twist1/TP (−/+), Twist1/TP (+/−), and Twist1/TP (+/+) according to the Twist1/TP expression levels. **a** Correlation between Twist1/TP expression and HCC characteristics, including metastasis, clinical stage, pathology grade, carcinoembryonic antigen (CEA) level, alpha-fetoprotein (AFP) level, and gender. **b** Overall survival analysis of Twist1/TP on patients with HCC. **c** Correlation analysis between vasculogenic mimicry (VM) formation and Twist1 and TP expression in 306 cases of HCC. The VM channel was PAS positive, but it did not express CD31 (red arrow). Endothelial vessels were PAS- and CD31-positive (yellow arrow). **d** Analysis of the HCC specimens by IHC. VE–Cad, vascular endothelial growth factor receptor 1 (VEGFR1), and VEGFR2 were minimally expressed in the Twist1- and TP-negative expression groups. When Twist1 and TP were individually or both positively expressed, the expression levels of the three marker proteins increased. **e** Statistical analysis of protein expression in 306 HCC specimens among the four groups (mean ± SD; *P < 0.05; **P < 0.01)

To clarify the promotion effect of the Twist1–TP transcriptional pattern on HCC VM formation and metastasis, we divided the 306 cases of clinical HCC specimens into four groups of Twist1/TP (−/−), Twist1/TP (−/+), Twist1/TP (+/−), and Twist1/TP (+/+) and analyzed the correlations between Twist1/TP and HCC characteristics. When TP was negatively expressed, malignant progression characteristics, namely, metastasis, clinical stage, pathology grade, CEA level, AFP level, total protein level, and survival time, were greatly affected even though Twist1 was positively expressed. Although Twist1 expression was negative in some samples, the high expression of TP significantly promoted the metastasis, clinical stage, and protein levels of CEA and AFP, which represent the malignant progression of HCC, and reduced the survival time of patients with HCC (Fig. 5a, b, Figure S2). The relation between Twist1/TP and VM formation was analyzed in our HCC clinical specimens by double labeling with CD31 and PAS. When Twist1 and TP were negatively expressed, the VM formation was very low (0.58 ± 0.13/high-power field (HPF)). The VM formation increased more than 9 times when only TP was positively expressed (4.71 ± 0.41/HPF) and nearly 12 times when Twist1 and TP were positively expressed (8.26 ± 0.40/HPF). The specimens with Twist1 expression but without TP expression exhibited VM formation of 3.07 ± 0.24/HPF (Fig. 5c). From the IHC results of the clinical HCC specimens, we found that the staining degrees of VE–Cad, VEGFR1, and VEGFR2 were higher in the Twist1 or TP staining positive groups than in the double-negative labeling group. A higher staining degree was observed when Twist1 and TP were positively expressed (Fig. 5d, e).

**Fig. 6:**
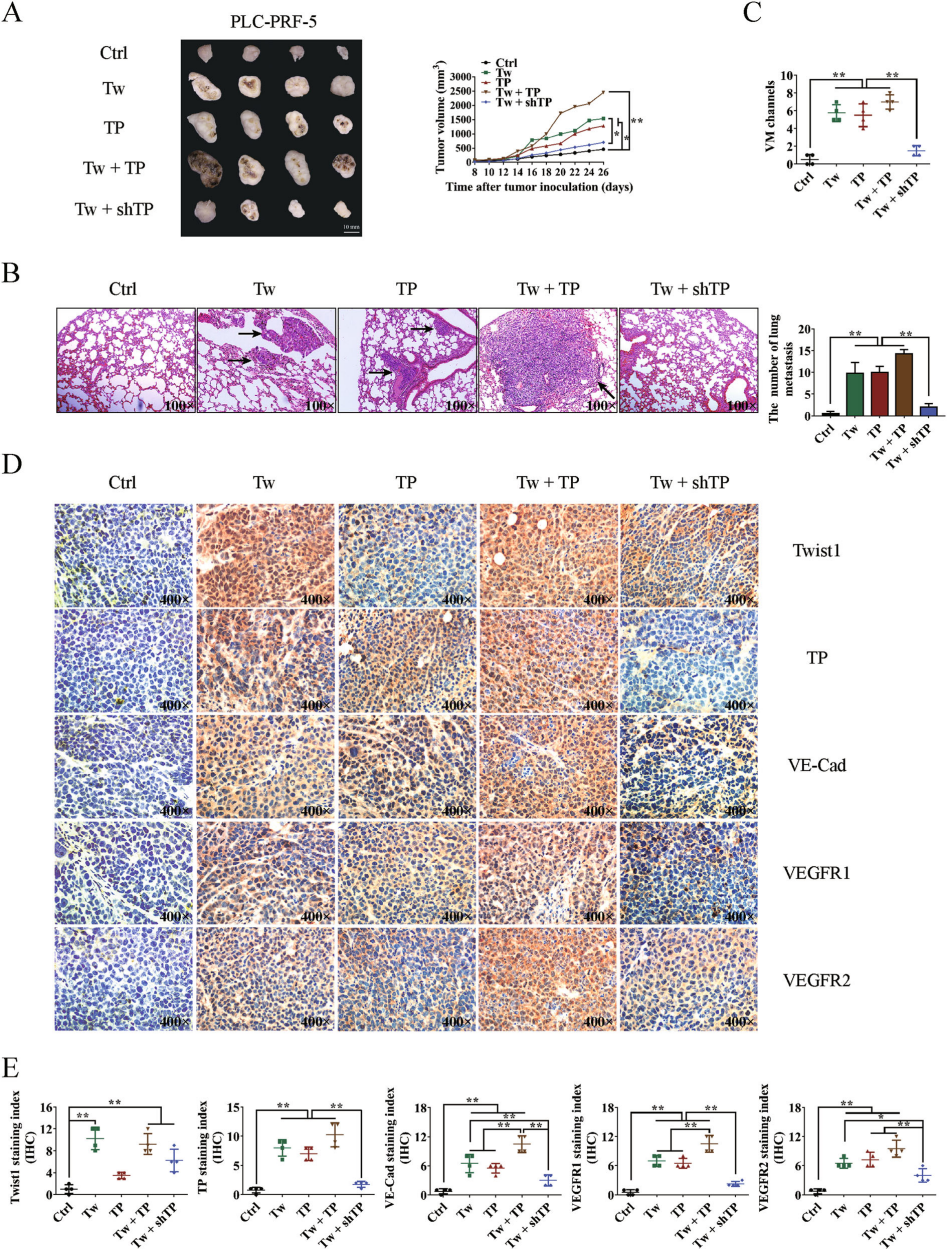
Effects of the transcriptional pattern of Twist1 to thymidine phosphorylase (TP) on hepatocellular carcinoma (HCC) growth, metastasis, and vasculogenic mimicry (VM) formation in the xenograft model. **a** Twist1 and TP promoted PLC-PRF-5 xenograft growth, whereas knocking down TP impaired the promotion effects of Twist1. **b** Number of tumors formed and lung metastasis in different groups. Twist1 and TP promoted lung metastasis of HCC, while knocking down of TP attenuated the promotion effect of Twist1. Images were taken at ×100 magnification. **c** Statistical analysis of VM formations in different groups. **d** Analysis of Twist1, TP, VE–Cad, vascular endothelial growth factor receptor 1 (VEGFR1), and VEGFR2 expression levels in xenograft tumors. Images were taken at ×400 magnification. **e** Statistical analysis of Twist1, TP, VE–Cad, VEGFR1, and VEGFR2 expression levels in different treated xenograft tumors (mean ± SD; *P < 0.05; **P < 0.01)

PLC-PRF-5 xenograft model experiments were conducted using Balb/c-nu/nu mice. When Twist1 and TP were individually or both overexpressed, tumor growth and lung metastasis were significantly enhanced. When TP was knocked down after overexpressing Twist1, the tumor volume and lung metastasis were impaired almost to the control level (Fig. 6a, b). We then measured VM formation through double staining with CD31 and PAS. The control group exhibited the fewest number of VM (0.50 ± 0.29/HPF) among the groups. After overexpressing Twist1 or TP, the VM formations increased to 5.75 ± 0.48/HPF or 5.50 ± 0.65/HPF, respectively. When Twist1 and TP were overexpressed, the VM formation further increased (7.00 ± 0.41/HPF). When Twist1 was overexpressed and TP was knocked down, the VM formation was reduced to almost the control level (1.50 ± 0.29/HPF) compared with the three other groups (Fig. 6c). Consistent with these results, the IHC analysis demonstrated that Twist1 and TP promoted VM marker expression and that TP knock down weakened the promotion effect of Twist1 overexpression (Fig. 6d, e).

### TP enzyme inhibitor suppresses HCC VM formation and metastasis

**Fig. 7:**
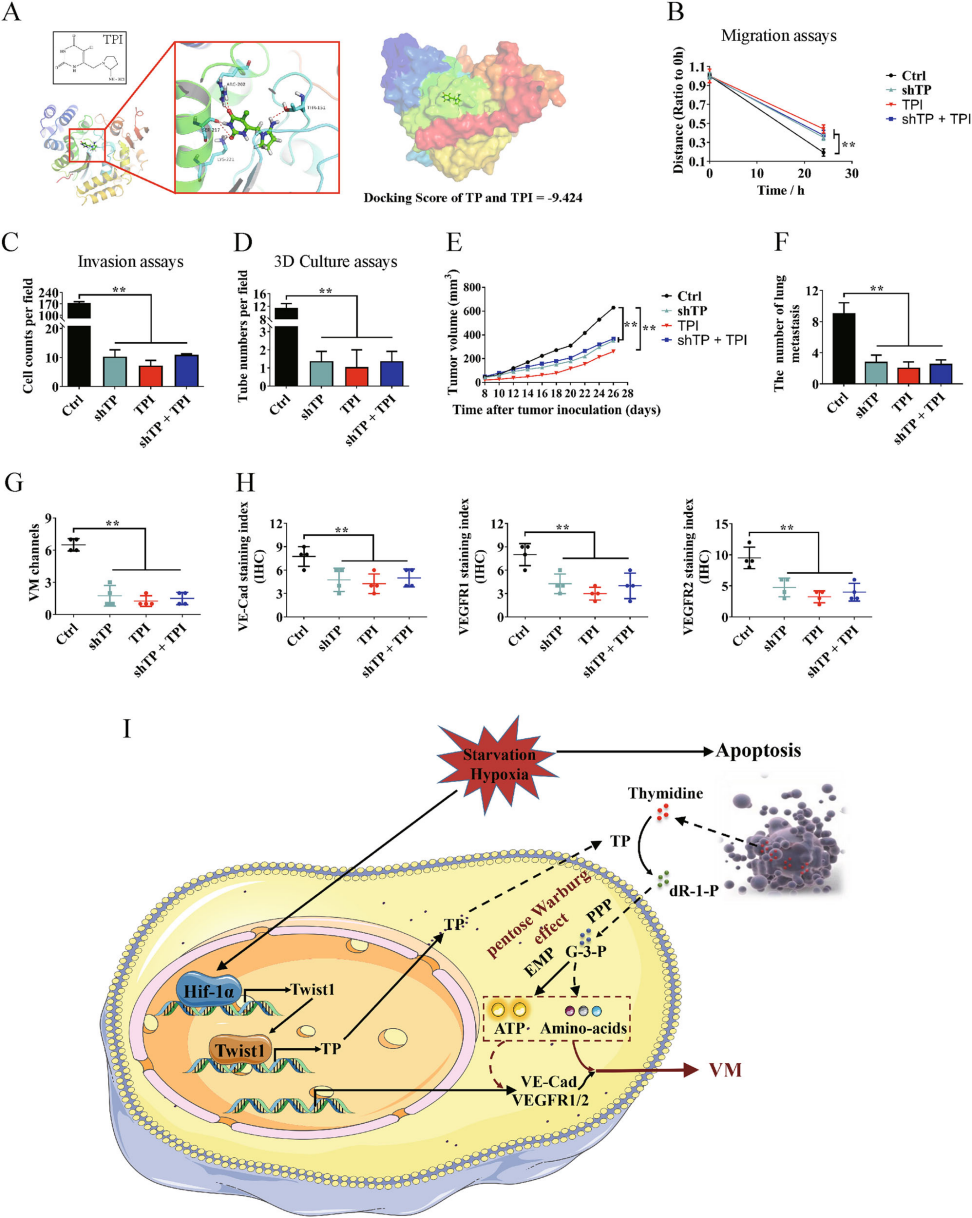
Effects of thymidine phosphorylase (TP) enzyme inhibitor on hepatocellular carcinoma (HCC) vasculogenic mimicry (VM) formation and metastasis. Hep3B cells were treated by knocking down TP and/or adding TP enzyme inhibitor tipiracil (TPI). **a** TPI exhibited good binding activity to TP determined by molecular docking. **b** Wound healing assay was performed. Quantitative analysis showed a significant difference in the speed of migration among different treated Hep3B cells. **c** Invasion assay showed a significant difference in the speed of invasion among different treated Hep3B cells. **d** Knocking down TP and/or adding TPI inhibited tube formation as detected by three-dimensional culture assay. **e** Effects of knocking down TP and/or adding TPI on Hep3B xenograft tumor growth. **f** Number of tumor formations and lung metastasis in different groups. Images were taken at ×100 magnification. **g** Statistical analysis of VM formation in different treated Hep3B xenograft tumors. **h** Statistical analysis of VE–Cad, vascular endothelial growth factor receptor 1 (VEGFR1), and VEGFR2 expression levels in different treated Hep3B xenograft tumors. **i** Proposed regulatory mechanism between Twist1–TP and VM in HCC. (mean ± SD; **P < 0.01)

TPI^25^ (Fig. 7a) was used to clarify the promotion effects of TP on HCC VM formation and metastasis and explore the possibility of using TP as a therapeutic target for HCC. When TP was knocked down in Hep3B cells, TPI exhibited weaker inhibitory effect on cell migration than the single addition of TPI (Fig. 7b, Figure S3A). Knocking down TP or adding TPI significantly inhibited Hep3B cell invasion and tube formation. The suppression when TP was knocked down and TPI was added was not as effective as when TPI was added alone (Fig. 7c, d, Figures S3B–C). A nude mouse model transplanted with Hep3B cells was established. TPI effectively inhibited the growth of xenograft HCC. When TP was knocked down, the inhibitory effect of TPI was reduced (Fig. 7e). Knocking down TP or adding TPI significantly inhibited lung metastasis, and the former weakened the inhibitory effect of TPI (Fig. 7f, Figure S3D). Finally, the VM formation and the expression of VM-related marker proteins were analyzed. TPI significantly inhibited the VM formation and decreased the expression levels of VE–Cad, VEGFR1, and VEGFR2. When TP was knocked down, the inhibitory effect of TPI weakened (Fig. 7g, h).

## Discussion

HCC is a common malignant tumor that causes high mortality worldwide. Although hepatic resection and liver transplantation have considerably improved the overall survival rate of patients with HCC, distant metastasis contributes to the failure of these treatments^26^. Ischemia and hypoxia caused by the deterioration of the local tumor microenvironment mainly induce tumor metastasis^27,28^. VM is an effective solution to the deterioration of the local tumor microenvironment^29^. Hypoxia-related problems could be solved by tumor cells through the activated hypoxia-inducible factor (HIF)-1α pathway^30^; however, the mechanism of carbon source selectivity of tumor cells remains unclear during glucose deprivation caused by ischemia. Stemness and EMT are regarded as major mechanisms in VM formation and metastasis and are associated with metabolic reprogramming^31,32^. The present study aims to determine the involvement of VM formation and metastasis in metabolic reprogramming. Our results showed that metabolic reprogramming driven by TP is related to VM formation and metastasis.

Twist1, as a transcription factor, has been identified as an important EMT inducer^33^. By TCGA data analysis and molecular assays, we demonstrated that Twist1 binds to two regions of the promoter sequences of TP and transcriptionally activates its expression. The negative correlation of TP and Twist1 in colon cancer might be due to different Twist1 transcriptional complexes^34^. However, the type of Twist1 complex formed must be further investigated. Vascular supply occlusion stimulates the expression of TP, which induces migration and angiogenesis in endothelial and tumor cells^16,17,35^. By catalyzing thymidine, TP can provide pentose for tumor cells^36^. TP was not a traditional secreted protein for not carrying a signal peptide sequence but could be secreted extracellularly by covalently linking to the phosphate groups of nucleotides through the serine residues of TP^37,38^. The present study investigated whether the secreted TP retains enzymatic activities, the relation of pentose generated from TP to the deterioration of the local tumor microenvironment, and whether TP is an important metabolic reprogramming protein in HCC VM formation and metastasis.

By analyzing the RNA-seq and metabolomics results, we found that, in the absence of glucose, the secreted TP can promote the generation of ATP in HCC cells and the survival of tumor cells by catalyzing extracellular thymidine. Further analysis revealed that TP activity promoted the conversion of pentose into G-3-P. Considering the lack of glucose and increase in the NADPH content, we concluded that the upgeneration of G-3-P did not occur through glycolysis and the normal pentose phosphorylate pathway. The normal Warburg effect describes a metabolism switch of incomplete oxidation of glucose even in the presence of oxygen in cancer cells^39^. This study indicated that tumor cells could convert pentose into G-3-P, which will enter into the glycolysis pathway; this process is therefore called the pentose Warburg effect. The results provide a further explanation of the mechanism of TP in affecting tumor metabolism^40^.

Further in vitro and in vivo experiments showed that Twist1 relied on TP activity to promote HCC metastasis and VM formation. The effects of TP on promoting VM formation gradually improved with increasing thymidine concentration. Considering that Twist1 can directly and transcriptionally activate VE–Cad, we explored whether TP could promote the stability or expression of Twist1 or its upstream regulatory proteins, such as HIF-1α^41^. However, no valid results were obtained (data not shown). As such, TP influenced the expression of VE–Cad through other pathways, and further explorations are needed. Finally, the TP enzyme inhibitor TPI was used in the experiment. The results confirmed the promotion effects of TP on HCC VM formation and metastasis and demonstrated the possibility of using TP as a therapeutic target in HCC.

In summary, this study described for the first time that TP was transcriptionally regulated by Twist1 and that TP activity promoted HCC metastasis and VM formation through the pentose Warburg effect under a deteriorated tumor microenvironment (Fig. 7i). When the local microenvironment deteriorated due to hypoxia and ischemia, Twist1 was first upregulated by HIF-1α and then it upregulated TP. The secreted TP converted extracellular thymidine into pentose, which was then used by tumor cells to generate energy through the pentose Warburg effect. Finally, the microenvironmental stress of tumor cells was alleviated, providing a basis for their malignant progression. This study demonstrated the dependence of Twist1 on TP to regulate tumor metabolic reprogramming and further enriched knowledge on the effects of Twist1 on tumor VM formation and metastasis. TP might be a potential therapeutic target in HCC. Our findings might benefit research on the mechanism of HCC malignant progression and provide new strategies for HCC diagnosis and therapy.

## Supplementary information

Supplemental Material
