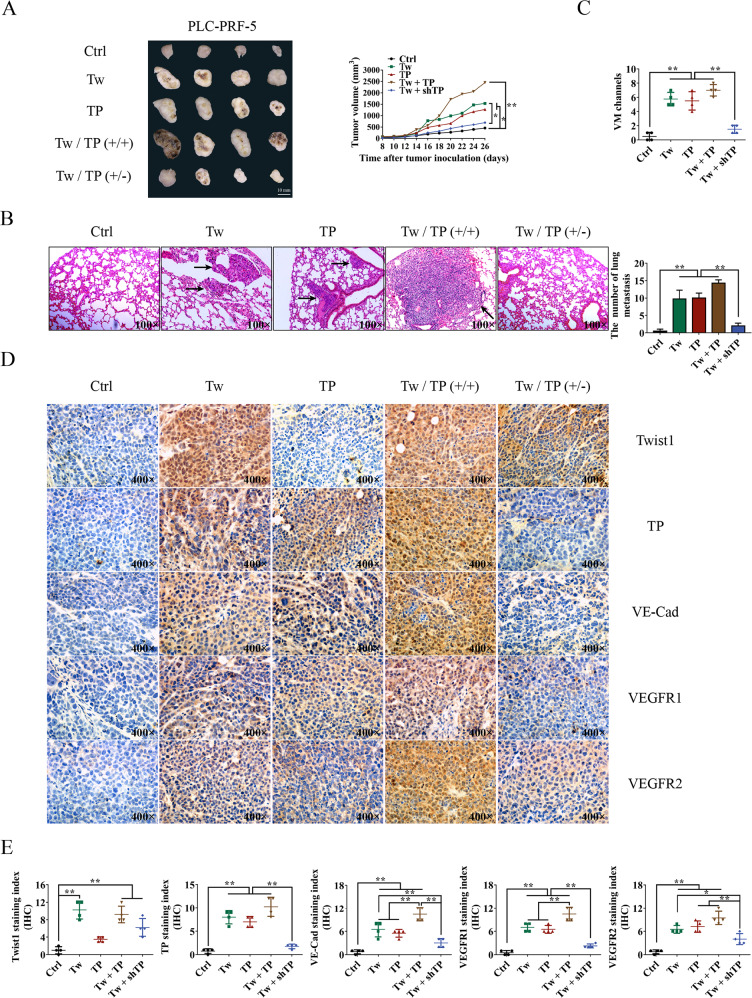# Correction: Thymidine phosphorylase promotes malignant progression in hepatocellular carcinoma through pentose Warburg effect

**DOI:** 10.1038/s41419-022-04557-7

**Published:** 2022-02-09

**Authors:** Qiang Zhang, Yuan Qin, Jianmin Zhao, Yuanhao Tang, Xuejiao Hu, Weilong Zhong, Mimi Li, Shumin Zong, Meng Li, Honglian Tao, Zhen Zhang, Shuang Chen, Huijuan Liu, Lan Yang, Honggang Zhou, Yanrong Liu, Tao Sun, Cheng Yang

**Affiliations:** 1 State Key Laboratory of Medicine Chemical Biology and College of Pharmacy, Nankai University, Tianjin, China; 2 Tianjin Key Laboratory of Molecular Drug Research, Tianjin International Joint Academy of Biomedicine, Tianjin, China; 3 Department of Pathology, Beijing Friendship Hospital, Capital Medical University, Beijing, China; 4 College of Life Science, Nankai University, Tianjin, China

**Keywords:** Metabolomics, Cancer metabolism, Tumour angiogenesis, Metastasis

Correction to: *Cell Death and Disease* 10.1038/s41419-018-1282-6, published online 17 January 2019

The original version of this article unfortunately contained a mistake in Fig. 6d. The authors pasted a wrong image from Group Twist1 + shTP (TP) for Group TP (Twist1). The coding for these two groups were similar, which made the authors paste a confused image before. This is a representative picture of immunohistochemistry. We examined the original data carefully. The statistical figures (Fig. 6e) showed the statistical results of the scores (average score of 4 visual fields in each section) of 4 pathological sections in each group. There is no problem with the data in the statistical figures. Although this does not affect the final conclusion, the authors apologize for the mistake. The revised figure can be found below.