# Supplementary material for: Thymidine phosphorylase promotes malignant progression in hepatocellular carcinoma through pentose Warburg effect
Source: Cell Death Dis. 2019 Jan 17;10(2):43. doi: 10.1038/s41419-018-1282-6 (PMC6426839; doi:10.1038/s41419-018-1282-6)
Supplement: Supplementary file 1 — Supplemental Material [file 41419_2018_1282_MOESM1_ESM.docx]

**Supplemental Figures**

**
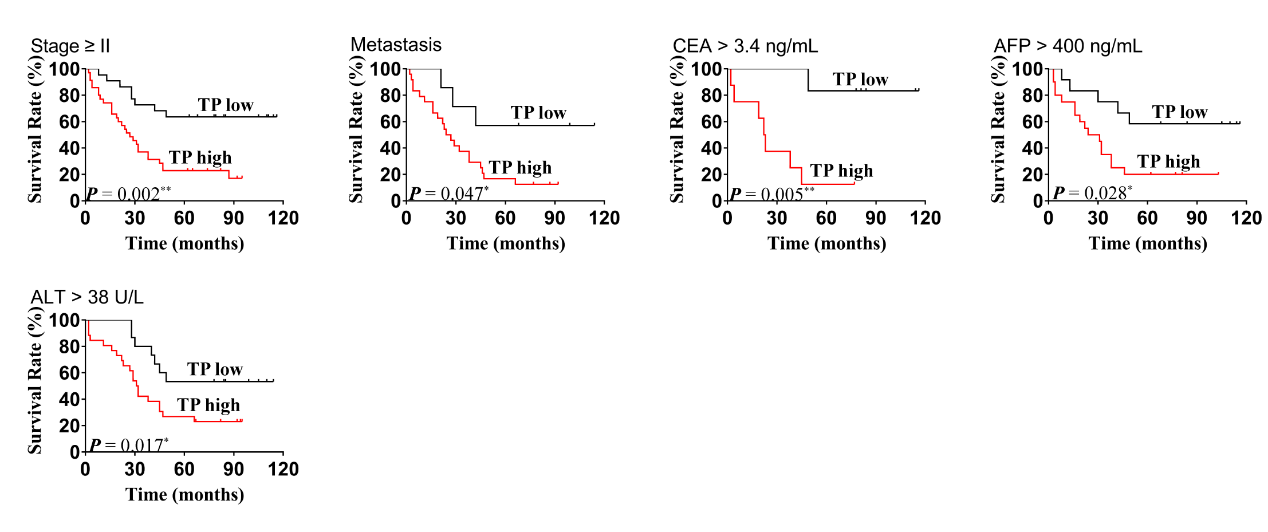
**

**Figure. S1**

Kaplan-Meier survival analysis results of TP expression significantly affected the survival prognosis of HCC patients with clinical parameters of stage ≥ II, or metastasis, or CEA content > 3.4 ng/mL, or AFP content > 400 ng/mL, or ALT content > 38U/L. (^*^P < 0.05; ^**^P < 0.01).

**
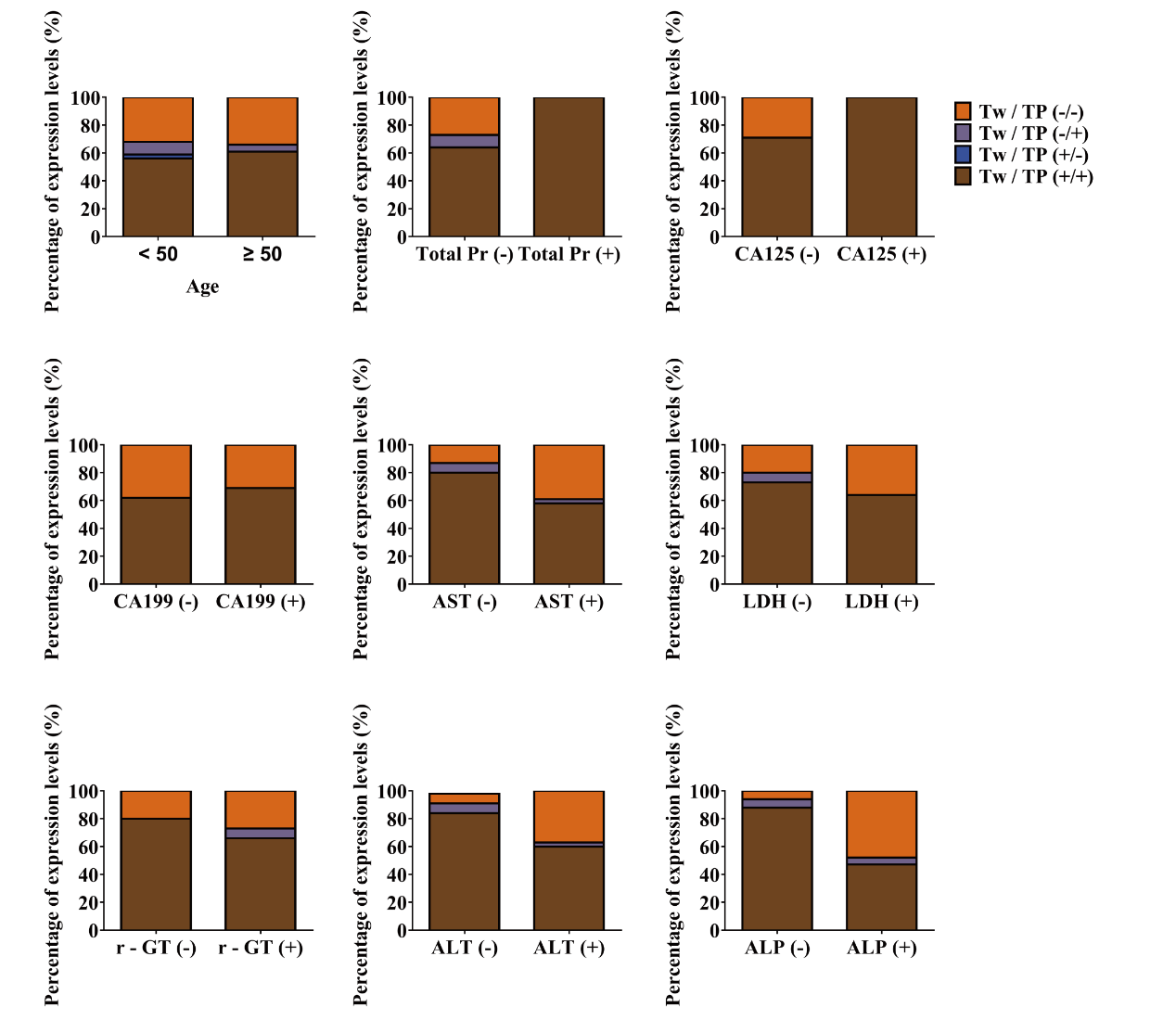
**

**Figure. S2**

Effects of Twist1/TP on clinical pathological characteristics of age, total protein level, CA125 level, CA199 level, AST level, LDH level, r-GT level, ALT level, and ALP level in HCC clinical specimens.

**
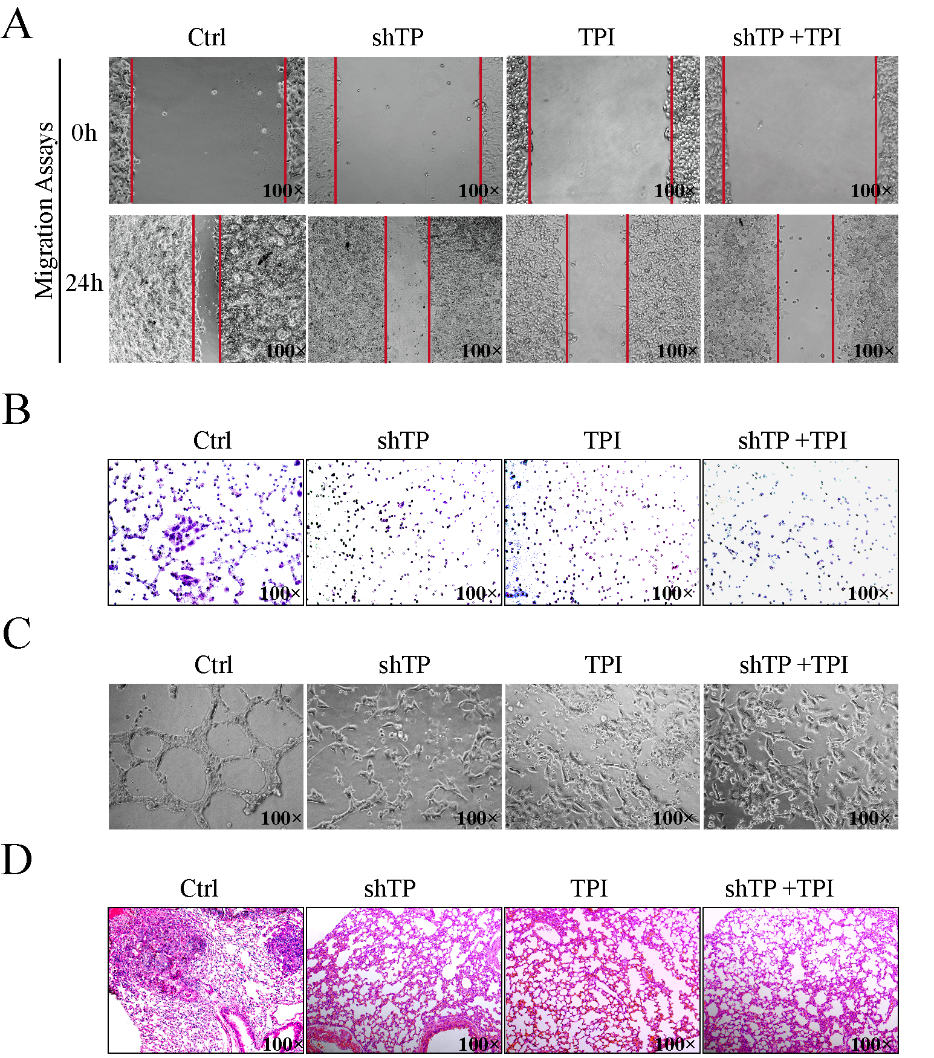
**

**Figure. S3**

**A.** Effects of TP enzyme inhibitor TPI on Hep3B cells migration. **B.** Effects of TP enzyme inhibitor TPI on Hep3B cells invasion. **C.** Effects of TP enzyme inhibitor TPI on Hep3B cells tube formation. **D.** Effects of TP enzyme inhibitor TPI on lung metastasis of Hep3B xenograft.

|  | | **PGL4.3-TP truncated primers** |
| --- | --- | --- |
| Full | F | 5’-CGGGGTACCGCGGAACAGCAGAG-3’ |
|  | R | 5’-GAAGATCTCGGCGTGGGTCTCGGGCGT-3’ |
| Region 1 | F | 5’-CGGGGTACCGCGGAACAGCAGAG-3’ |
|  | R | 5’-GAAGATCTCGGACCCAAGAGCGAGGGC-3’ |
| Region 2 | F | 5’-CGGGGTACCCGCGTCATCGGCAAAGT-3’ |
|  | R | 5’-GAAGATCTCTCACGACCTGATAGGCGC-3’ |
| Region 3 | F | 5’-CGGGGTACCTCCAGGGGTCTACAAGT-3’ |
|  | R | 5’-GAAGATCTCTGGACCCCCGGGGACTCT-3’ |
| Region 4 | F | 5’-CGGGGTACCGGGTCCCGGCGCGGAG-3’ |
|  | R | 5’-GAAGATCTCACGATGCGGACTCGCAGACT-3’ |
| Region 5 | F | 5’-CGGGGTACCGTCTGCGAGTCCGCATCGTGT-3’ |
|  | R | 5’-GAAGATCTCGGCGTGGGTCTCGGGCGT-3’ |
|  | | **PGL4.3-TP mutated primers** |
| Motif1M | F | 5’-CGGGGTACCGCGGAACAGCAGAG-3’ |
|  | M1L | 5’-CCTTGACCCTGGGCTACGTTGTAGGCCGCGGGCCC-3’ |
|  | M1R | 5’-CGCGGCCTACAACGTAGCCCAGGGTCAAGGGTCAG-3’ |
|  | R | 5’- GAAGATCTCGGCGTGGGTCTCGGGCGT -3’ |
| Motif2M | F | 5’-CGGGGTACCGCGGAACAGCAGAG-3’ |
|  | M2L | 5’-GACTCGCAGACTTTAGCCGCTCCCGCCTGGCGGGT-3’ |
|  | M2R | 5’-CCAGGCGGGAGCGGCTAAAGTCTGCGAGTCCGCAT-3’ |
|  | R | 5’-GAAGATCTCGGCGTGGGTCTCGGGCGT-3’ |

**Supplemental Tables**

**Table S1**

**PGL4.3-TP primers:**
